# Computational modeling of intravitreal ranibizumab kinetics: Predicting macular drug concentration and half-life

**DOI:** 10.1371/journal.pone.0348811

**Published:** 2026-05-07

**Authors:** Jabia Mostofa Chowdhury

**Affiliations:** Department of Electrical Engineering, Texas A&M University–Texarkana, Texarkana, Texas, United States of America; Sanmenxia Central Hospital, Henan University of Science and Technilogy, CHINA

## Abstract

Ranibizumab is a key anti-VEGF therapeutic used to improve treatment efficacy and reduce injection frequency in neovascular retinal diseases. Because experimental pharmacokinetic data in humans are limited, computational modeling provides an effective means to predict ocular drug behavior. In this study, a three-dimensional computational model of the human eye was developed in COMSOL Multiphysics 6.3 to analyze the pharmacokinetics of ranibizumab following intravitreal injection. The model also quantified drug concentration near the macula, the primary target site for neovascular disease, and evaluated it against the minimum threshold concentration required for VEGF suppression. Fluid flow in the vitreous was represented by Darcy’s law, while drug convection and diffusion were modeled using the transport of diluted species equation. Four physiological scenarios combining vitreous state (normal vs. partially liquefied) and elimination pathways (anterior dominant vs. both routes) were simulated. Predicted intravitreal half-lives ranged from 2.7 to 4.4 days, consistent with reported human data (2.4 ~ 9 days). The model demonstrates a plausible representation of physiologically relevant ocular pharmacokinetics and provides a computational framework that may assist in exploring dosing strategies and informing the design of intravitreal drug delivery systems.

## Introduction

Neovascular eye diseases, including age-related macular degeneration (AMD), diabetic retinopathy (DR), and retinal vein occlusion (RVO), are characterized by abnormal blood vessel growth driven by overexpression of vascular endothelial growth factor (VEGF) [1,2]. These conditions represent a significant and growing public health concern, affecting millions of individuals worldwide. There are 11 million Americans who are currently suffering from AMD, and the number is expected to double by 2050 [3]. Statistics showed that over 100 million people around the world are impacted by diabetic retinopathy, where RVO is considered the second most common retinal vascular disease, impacting 16 million adults globally [4].

The treatment of neovascularization is largely driven by retinal ischemia and oxidative stress, which trigger a hypoxic condition that is responsible for VEGF production [5,6]. VEGF promotes endothelial cell proliferation and migration, leading to the development of fragile, leaky blood vessels that are susceptible to hemorrhage and exudation [7–9]. This cascade ultimately results in retinal edema and progressive vision loss [10].

The development of choroidal neovascularization in non-exudative AMD is also driven by chronic inflammation [2,11,12]. The persistent inflammatory environment associated with drusen formation and retinal pigment epithelium (RPE) dysfunction plays a key role in upregulating VEGF expression [13]. Diabetic retinopathy involves similar underlying mechanisms, where chronically elevated blood glucose levels in diabetic patients cause progressive microvascular damage and capillary blockage. This results in secondary retinal ischemia, which in turn stimulates the release of VEGF [6]. The central role of VEGF in these neovascular pathologies is well supported by clinical evidence, forming the rationale for anti-VEGF therapies such as ranibizumab and bevacizumab [1].

Ranibizumab and bevacizumab are two widely used anti-VEGF (vascular endothelial growth factor) agents with similar purposes but differing physical properties [14–16]. Ranibizumab is an FDA-approved antibody antigen-binding fragment (Fab) that inhibits VEGF-A within the intraocular space, which is used to reduce the rate of diabetic retinopathy in patients with diabetic macular edema (DME)and AMD [17,18]. Key pharmacokinetic differences stem from their molecular size: the smaller Fab fragment ranibizumab (~48 kDa) penetrates the retina more rapidly (within ~1–3 days) than full-length bevacizumab (~149 kDa, ~ 7 days to retinal penetration). Consistently, ranibizumab also exhibits a shorter vitreal half-life (~2.9 days) compared to bevacizumab (~4.3 days) [14,19].

Given the complex nature of neovascular diseases, multiple treatment modalities have been explored, including eye drops, near-infrared light (NIR) therapy, port delivery systems (PDS), oral or intravenous administration, and intravitreal injections. In neovascular eye disorders like AMD and diabetic retinopathy, anti-VEGF therapy plays a pivotal role, and intravitreal injections provide agents such as Ranibizumab and Bevacizumab with direct access to retinal tissue, ensuring maximum efficacy with minimal systemic exposure. One notable drawback of ranibizumab’s small size is limited retention in the sclera (minimal scleral depot effect), potentially shortening its duration of action after intravitreal injection [19].

In this study, we develop a three-dimensional pharmacokinetic (PK) model of the human eye to simulate the spatial and temporal distribution of ranibizumab following intravitreal injection. The model incorporates relevant anatomical parameters and physiological elimination pathways to describe drug transport within the vitreous humor. Using this computational framework, we estimate ranibizumab concentrations both in the vitreous and at the macular region, the primary target site of neovascular pathology. As experimental PK data for ranibizumab in the human vitreous are unavailable, computational data are used to assess the plausibility and consistency of the present PK results [20]. In addition, we analyzed our model consistency by comparing the predicted vitreal half-life with established literature values. Finally, we determine the effective duration of ranibizumab based on concentration thresholds derived from in vitro reference data, corresponding to VEGF suppression in the macular region.

Several three-dimensional PDE-based models of intravitreal anti-VEGF transport have been reported previously. These studies have provided important insights into global vitreous pharmacokinetics, alternative delivery routes, and PK–PD mechanisms. In contrast, the present work focuses specifically on spatially resolved drug concentrations at the macular region, the primary therapeutic target in neovascular retinal disease. By applying therapeutic concentration thresholds at the macula, this model enables estimation of effective treatment duration at the target site, complementing prior vitreous-centered pharmacokinetic analyses.

## Methods

### Vitreous humor

Vitreous humor is a colorless, viscoelastic gel-like substance that is the largest part of the eye. Interestingly, no blood vessels are present in this part; rather, its gelatinous consistency is attributed to the presence of hyaluronic acid and collagen [21–23]. Aging is one of the key factors that cause significant biochemical and structural changes in the vitreous, including vitreous liquefaction and collagen fiber aggregation, which play a critical role in the pathogenesis of various vitreoretinal disorders [24].

The liquefied portion in the vitreous humor forms a pocket of liquid within the gel. The location of the liquefied pocket in the vitreous depends on various factors like ocular movements, vitreous degeneration, and age. For modeling simplicity, the shape of the liquefied region is often idealized as spherical [23,25]. In recent years, several computational studies have modeled the effect of partially liquefied vitreous humor with [23,24] and without [26,27], considering the influence of eye movements, providing insights into ocular drug delivery, disease progressions, and the mechanical behavior of the eye. In this paper, we simulated four distinct scenarios combining two vitreous conditions and two elimination route assumptions, as summarized in Table 1.

**Table 1:** Summary of case studies.

	Partially liquefied VH	Anterior elimination	Both elimination
Study 1	√	X	√
Study 2	√	√	X
Study 3	X	X	√
Study 4	X	√	X

In our study, we investigated the intravitreal administration of the drug ranibizumab transport in the vitreous humor. The intravitreal injection location is an important parameter in drug distribution and influences the vitreous half-life calculation. Experimental studies have shown that ranibizumab is typically injected 1–4 mm behind the surgical limbus region using a 30-gauge needle into the vitreous cavity [17,28,29]. In our model, we have fixed the injection site in the anterior vitreous at the Cartesian coordinate (0, 6, 3) mm, which represents the clinically standard anterior–supertemporal location relative to the vitreous center defined at (0, 0, 0) mm.

The elimination of the drug through the vitreous humor occurs via two major routes: anterior and posterior. Several studies have shown that the drug ranibizumab is eliminated through the aqueous humor outflow [28,30]. Considering the significant influence of the anterior elimination route, our model explores both anterior and posterior dominant elimination routes separately. Table 1 includes a summary of the case studies we investigated in our model.

We investigated four combinations of studies in our model:

- **Study 1:** Partially liquefied vitreous + Both anterior and posterior elimination routes.
- **Study 2:** Partially liquefied vitreous + Anterior dominant elimination
- **Study 3:** Normal (non-liquefied) vitreous + Both elimination routes.
- **Study 4:** Normal vitreous + Anterior dominant elimination only.

The partially liquefied (PL) vitreous geometry was based on the model by Khoobyar et [26], whereas the “normal” vitreous had no liquefied region. By comparing these cases, we examined the impact of vitreous liquefaction and elimination pathways on ranibizumab kinetics.

### Geometry model

**Fig 1:**
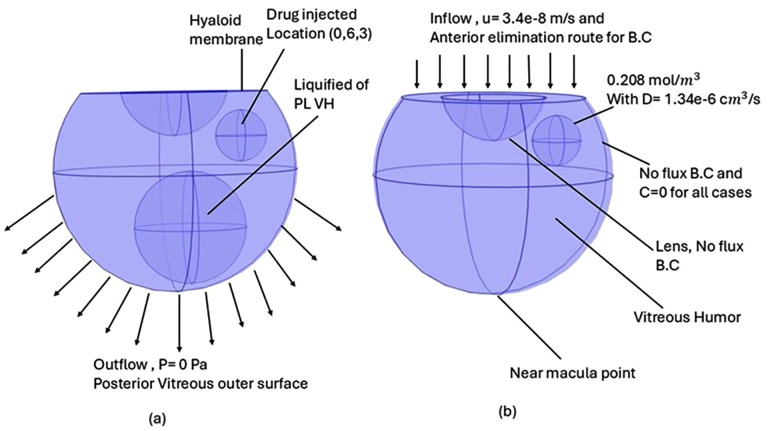
3D geometry of the human eye with anatomical regions and boundary conditions (B.C) (a) Partially liquefied (PL) VH and (b) VH without PL.

A realistic geometric model is an essential component of mathematical modeling, which should be physiologically and anatomically accurate to yield more realistic simulation results that can be validated against experimental data. In this paper, we constructed a 3D model of the human vitreous chamber with physiologically appropriate dimensions, as shown in Fig 1. The three-dimensional geometric models of the human vitreous were constructed as spheres in COMSOL. Studies suggested the range of volume of human vitreous humor varies from 4mL to 5.43mL [31, 32]. The volume of the vitreous chamber in our model is 4.79 mL, located in a 3D spherical domain with origin $\left(x_{v},y_{v},z_{v})=(\text{0},\text{0},\text{0}\right)$ mm. The retina, lens, and hyaloid membrane are three major tissues that are bound to the vitreous humor. The radius of the human vitreous in our model is 10.9 mm. The vitreous outer surface is divided into anterior and posterior, where the hyaloid membrane works as a separator between the aqueous humor and the vitreous humor in our model. The radius of the lens is 4.9 mm. The parameter details of our 3D human eye geometry are adapted from our article, Chowdhury, Jabia et al. [33]. In the partially liquefied vitreous scenario, a spherical liquid cavity of radius 5 mm was incorporated in the mid-vitreous, following the geometry used by Khoobyar et al. [26].

#### Fluid flow through vitreous humor.

Our vitreous humor is mostly porous under normal conditions, and Darcy’s law, used to model fluid flow in the vitreous, can be applied as:


$$u=\left(\frac{\kappa }{\mu }\right)\nabla p$$ (1)

Where u is the flow velocity, κ is the permeability of vitreous/ Darcy’s coefficient of the vitreous, where μ refers to the dynamic viscosity of the vitreous, $\frac{\kappa }{\mu }$ is defined as hydraulic conductivity, and p is defined as pressure.

The continuity equation:


$$\frac{\partial }{\partial t}(\rho \epsilon )+\nabla .(\rho u)=Q_{m}$$ (2)

Where, ρ is the density of the fluid, ε is the porosity of the vitreous humor.${\text{Q}}_{\text{m}}$is the mass source term which is set to zero. In our model, the porosity and the reference pressure of the vitreous are fixed, ∈=1 and p = 1atm respectively.

The fluid that enters the vitreous from the aqueous humor through the hyaloid membrane at a constant production rate of 3 μL/min. In our model, the hyaloid membrane is considered as an inlet velocity, where $u_{in}=$ 0.0000340 mm/s [33]. The fluid exits through the retinal boundary, considered as the outlet where, $P_{out}=$ 0 Pa [26].

### Fluid flows through partially liquefied vitreous

The Darcy-Darcy system is used to simulate a partially liquidized vitreous. Both regions, the liquid and gel regions, are governed by Darcy’s law. The Darcy’s coefficient (K) of the liquid region ($K_{L}$) considered thousands of times higher than the non-liquefied zone ($K_{G}$). For this model, Darcy’s law is used as:


$$u_{L}=\left(\frac{\kappa_{L}}{\mu }\right)\nabla P_{L}$$ (3)

$$u_{G}=\left(\frac{\kappa_{G}}{\mu }\right)\nabla P_{G}$$ (4)

Previous studies [26,27] influence the ratio of the liquid and gel-type material in our model: $\frac{K_{L}}{K_{G}}$ = ${\text{1}\text{0}}^{\text{3}}$ *where* G represents the gel-type region. The value [26] of hydraulic conductivity is fixed at: $\frac{K_{L}}{\mu }$ = $\text{9}\times {\text{1}\text{0}}^{-\text{6}}\frac{{mm}^{\text{2}}}{Pa.s}$. The other parameters used in this model are the same as normal vitreous condition.

#### Drug transport.

Our computational model of drug ranibizumab kinetics captured through the vitreous humor for both models: partially liquefied and normal vitreous conditions, is solved by the convection-diffusion-reaction equation:


$$\frac{\partial C}{\partial t}=\nabla .(D\nabla C)-u.\nabla C+R$$ (5)

Where C(x,t) is the concentration of the drug, D is the diffusion coefficient of the drug, u is the velocity of the fluid(from Darcy’s law), and R is the reaction rate, which is negligible in our model (R = 0). Both convection and diffusion were included in all simulations, as fluid flow can significantly influence intravitreal drug distribution in the vitreous (both conditions).

Our Pharmacokinetics model calculates drug concentration in the vitreous humor and near the central retinal point of the retinal layer. The drug ranibizumab distribution depends on the diffusion coefficient [34] rate, D = 1.34$\times {\text{1}\text{0}}^{-\text{6}}{cm}^{\text{2}}$/ s. The effect of drug metabolism and degradation of ranibizumab is considered negligible in our studies [15]. The drug ranibizumab dose for neovascular disease, like neovascular age-related macular degeneration, macular edema, and myopic choroidal neovascularization, is 0.5 mg/0.05mL and is recommended as an intravitreal injection [35]. We used the same amount of drug in our model: $C_{j}=\text{0}.\text{2}\text{0}\text{8}\text{3}\left[\frac{mol}{m^{\text{3}}}\right]$, where the radius of intravitral drug bolus, r = 2.33 mm [0.05 mL]

The initial drug concentration of ${\text{C}}_{\text{0}}$in the domain defined as:


$$C_{\text{0}}=\left\{ \begin{array}{c} @lC_{j}forr^{\text{2}}\\ \text{0}forotherwise \end{array}\right.$$ (6)

### Boundary condition

The vitreous humor is connected to three major tissues: the retina, lens, and hyaloid membrane. The anterior part of the vitreous and the lens are considered impermeable to fluids and drugs. No fluid or drug can pass through this tissue, for that zero velocity (u = 0) and no-flux boundary condition is applied.

The two main routes for drug elimination from the vitreous are the anterior route, defined by the hyaloid membrane, and the posterior route, which occurs through the blood-retinal layer. The pressure, fluid velocity, and drug concentration need to be measured within the vitreous chamber.

Both the models, the normal vitreous model and the liquefied vitreous model, follow the same boundary conditions. The no flux boundary condition is applied in the fluid flow is defined by:


−n.(ρu)=0 (7)

Where, ρ is the vitreous, u is the fluid velocity, and n is the normal vector term.

The initial value of the drug is set, c = 0, for all conditions. The no-flux boundary condition is used in drug transport governed by:


−n.(−D∇C+uC)=0 (8)

In this equation, D is the diffusion coefficient of the drug, n is the unit normal vector, C is the drug concentration, and u is the fluid velocity. The outflow of the drug transport is defined by:


$$-\text{n}.D_{i}\nabla c_{i}=\text{0}$$

The drug elimination route is considered through the boundary condition depicted in the geometry. Four different studies are performed considering the elimination route. In anterior elimination, the hyaloid membrane is the dominant drug elimination route. In both elimination cases, the posterior portion surrounded by the retinal layer of the vitreous is considered the major elimination route. To bracket physiologically plausible clearance behavior, we consider two limiting cases: a zero-concentration boundary (C=0), corresponding to infinitely fast removal, and a no-flux condition.

The zero-concentration boundary represents an upper limit on clearance, assuming instantaneous drug elimination, while the no-flux condition reflects the opposite extreme with negligible removal. Together, these bounds capture the likely range of physiological, permeability-limited clearance across ocular tissues [9]. Although actual elimination lies between these extremes, using both scenarios helps assess how different pathways influence ranibizumab pharmacokinetics. Studies 1 and 3 better reflect clinical conditions with combined anterior and posterior elimination, whereas Studies 2 and 4 emphasize anterior-dominated clearance.

### Computational simulation and model consistency analysis

The three-dimensional geometric model was created, and the simulation was done in COMSOL 6.3 Multiphysics. The Darcys’ law of physics was used to solve the vitreous humor simulation under normal and PL conditions. The drug transport through both the vitreous humor, considering two different elimination routes, was modeled using a transport-diluted species interface. The physics-controlled mesh in COMSOL was used to run the model.

The therapeutic objective of ranibizumab is to suppress vascular endothelial growth factor (VEGF) activity in the macula. Based on in vitro data, an efficacy threshold of 0.12 µg mL⁻¹ was adopted for VEGF neutralization [18,36]. During simulations, we recorded the duration for which the ranibizumab concentration at the macula remained at or above this threshold, as an indicator of how long the drug might be therapeutically effective after a single injection.

To further establish model credibility, qualitative spatial validation of the vitreous flow field was performed. An additional flow configuration was implemented using an inlet velocity of ${\text{u}}_{\text{in}}=\text{1}.\text{47}\times {\text{10}}^{-\text{4}}$mm/s, consistent with Darcy-based vitreous flow models, while all other parameters were unchanged. Localized vitreous liquefaction was modeled with a radius of r=2mm and r=5mm, following Khoobyar et al. (2022) [26]. Mid-plane pressure contours and Darcy streamlines were examined for localized liquefied regions of varying size and location. The spatially averaged drug concentration within the vitreous was obtained using a domain probe, and the macular concentration was extracted with a point probe located near the foveal center at coordinates [0, 0, –10.9 mm], corresponding to the central retina in the model geometry. In our study, we assessed model plausibility by comparing the predicted half-life of the intravitreal drug ranibizumab in the human vitreous. To estimate the intravitreal half-life of ranibizumab from the model, we noted the time at which the volume-averaged vitreous drug concentration declined to 50% of its initial value. This model-predicted half-life was then compared with published values from human studies as a form of consistency analysis.

Sensitivity analyses were performed by varying key transport parameters over one order of magnitude above and below baseline values. Specifically, the diffusion coefficient (D) and vitreous hydraulic conductivity (K) were varied as 0.1× , 1× , and 10× their nominal values. For each case, time-dependent drug concentration was evaluated in the vitreous humor and macular region, including half-life calculation. To verify the robustness of macular concentration measurements, drug concentration was evaluated using both a point probe and a finite-volume region of interest (ROI). The ROI was defined as a cylindrical domain with radius 1 mm and height 1 mm, attached to the retinal layer at the macular location. Time-dependent concentrations obtained from the ROI-averaged domain and point probe were compared.

## Results

**Fig 2:**
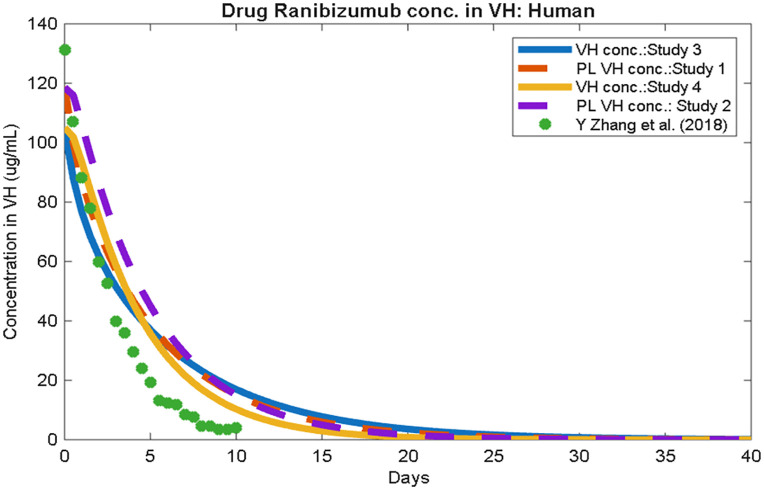
Vitreous concentration–time profiles for ranibizumab under four studies, compared with computational data from Zhang et al. (2018) [20].

Our pharmacokinetic study compares the ranibizumab concentration profiles in the vitreous for all four modeled scenarios, summarized in Fig 2. We attempted to investigate the impact of vitreous modeling on the drug transport mechanism in human vitreous. The results provide a controlled assessment of the extent to which partial liquefaction alone alters intravitreal pharmacokinetics. The simulations did not identify substantial differences between partially liquefied and normal vitreous models under the static conditions considered. The objective of the present analysis was to establish a direct comparison between normal and partially liquefied vitreous humor under identical anatomical geometry and boundary conditions, rather than model liquefaction incorporating saccadic motion effect. Under these static conditions, only modest changes in ranibizumab concentration profiles and half-life were observed.

We compared the predicted concentration-time profiles with the computational study reported by Zhang et al. (2018) to assess physiological plausibility [20].

The mechanisms governing intravitreal drug transport are complex and depend on multiple variables, including drug diffusivity, vitreous structure, boundary conditions, and individual ocular anatomy. The study found that the half-life of ranibizumab varies from 2.4 to 9 days, depending on the neovascular diseases and intravitreal treatment types. Published studies report that the population-based pharmacokinetic study shows the half-life of the drug ranibizumab is 7.2 ~ 9 days [34,36–39]. However, other approaches reported a vitreous half-life of ~9 days, while a model-based estimate suggested ~4.75 days [40]. A recent review by Daniele et al. summarized findings from multiple studies and estimated the half-life to be ~ 7.19 days [14]. Another study that calculated half-life using a mathematical model claimed their calculation found 4.75 days [35].

The half-life values reported here are based on volume-averaged vitreous concentration and are used for comparison with existing pharmacokinetic studies. These values represent global drug clearance behavior and do not directly reflect local drug dynamics at the macula. Our model-predicted ranibizumab half-lives for the four scenarios are summarized in Table 2.

**Table 2:** Calculated ranibizumab vitreous half-lives in days.

Conditions	Half-life in days
Study 1	2.8 days
Study 2	4.4 days
Study 3	2.7 days
Study 4	4.3 days

The values ranged from approximately 2.7 to 4.4 days. Notably, the cases assuming both elimination (Study 1 and 3) yielded shorter half-lives (2.7 ~ 2.8 days) than the cases with anterior dominant elimination (Study 2 and 4: 4.3 ~ 4.4 days). This result suggests that incorporating the anterior elimination route effectively slows overall drug clearance, leading to a longer retention of the drug in the eye. All four predicted half-lives fall within the broad range reported in the literature (2.4 ~ 9 days) and are particularly close to some model-based estimates. This agreement with published values supports the computational consistency of our model.

We also examined the influence of intravitreal injection location on ranibizumab transport by simulating three anatomically distinct sites within the vitreous: an anterior–supertemporal location near the limbus (0,6,3)mm, a mid-vitreous location (1.5,0,−3)mm, and a posterior location near the retina (0,0,−7)mm (coordinates in mm, with the vitreous center at (0,0,0)mm and +z oriented posteriorly toward the retina). It is important to note that while the posterior and mid-vitreous locations provided valuable data on transport sensitivity, these are not standard clinical injection sites and were used here solely for computational comparison.

Table 3 includes the predicted intravitreal half-lives for all the study cases at different injection locations. The results indicate that injection location can lead to measurable differences in ranibizumab clearance behavior, with posterior injections generally exhibiting shorter half-lives in some scenarios, while mid-vitreous or anterior injections showed longer persistence depending on elimination pathway and vitreous state.

**Table 3:** Predicted intravitreal half-life of ranibizumab for different intravitreal injection locations.

Injection Location	Anterior (0,6,3)	Middle (1.5,9,-3)	Posterior (0,0,-7)
Study 1	2.8 days	2 days	1.7 days
Study 2	4.4 days	4.5 days	3 days
Study 3	2.7 days	1.5 days	2 days
Study 4	4.3 days	4.8 days	4.6 days

**Fig 3:**
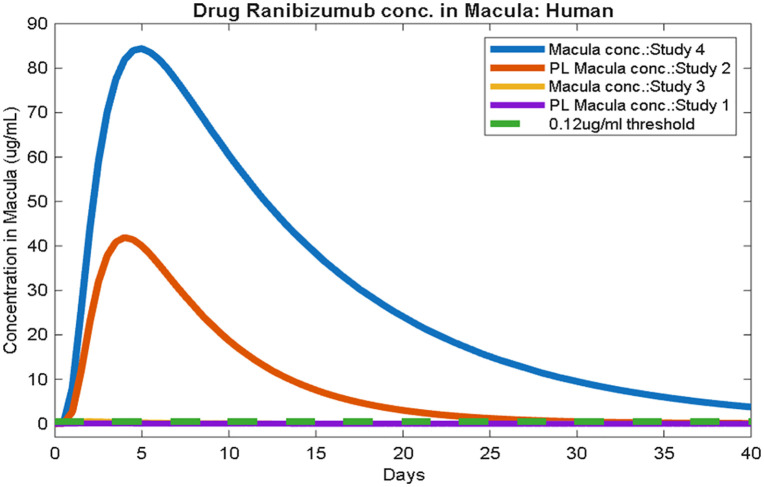
Drug concentration in the human macula and VEGF-suppression threshold.

We tracked the ranibizumab concentration at the macula (central retina) to assess how long it remained above the in vitro-derived therapeutic threshold of 0.12 µg/mL [36], which has been used as a reference concentration for VEGF neutralization. Figure 3 illustrates the macular concentration over time for each scenario, with the 0.12 µg/mL line for reference. Macular concentration represents a localized metric relevant to therapeutic efficacy, particularly for VEGF suppression, and is evaluated independently of the global half-life estimates. However, this threshold is derived from in vitro studies and may not directly represent the exact therapeutic requirement *in vivo* due to the complex physiological and biochemical environment of the human macula.

Macular concentration declines faster in both elimination cases (Studies 1 and 3) and falls below 0.12 µg/mL sooner than in the dual-route cases. In the simulations with anterior dominant elimination routes (Study 2:PL vitreous, and Study 4: normal vitreous), ranibizumab levels at the macula remained above 0.12 µg/mL for a longer duration compared to both elimination scenarios. These results indicate that inclusion of the anterior elimination pathway, while prolonging overall half-life, may also help sustain therapeutically effective ranibizumab levels at the macula for an extended period following injection.

**Fig 4:**
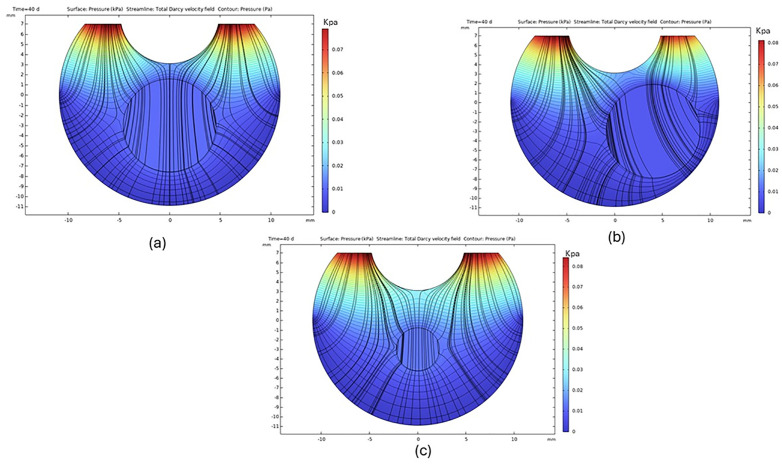
Pressure distribution and Darcy flow streamlines in the vitreous humor. The liquefied region is a sphere for (a) radius r=5mm located at (2,0,−3)mm, (b) radius r=5mm located at (−1,4,−3)mm, and (c) radius r=3mm located at (−1,4,−3)mm.

Figure 4 shows pressure contours and Darcy velocity streamlines within the vitreous humor for localized liquefied regions of partially liquefied vitreous at different sizes and locations. Simulations were performed using a liquefied region radius of r=5mm at two spatial locations to enable direct comparison with the reference model of Khoobyar et al. (2022) [26], as well as a smaller liquefied region with r=3mm. The results demonstrate distortion of pressure isobars and preferential flow focusing through the liquefied region. These spatial flow patterns are in good qualitative agreement with previously reported Darcy–Darcy vitreous flow models [26], with minor differences in peak pressure magnitude attributed to variations in three-dimensional geometry and flow field configuration.

**Fig 5:**
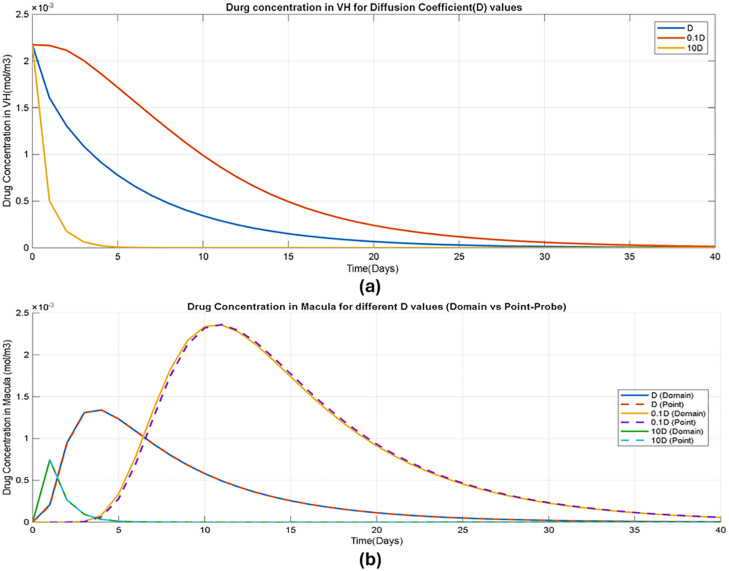
Sensitivity of drug concentration to diffusion coefficient (D) (a) Drug concentration-time profile in vitreous humor (b) Drug concentration-time profile in macula using domain vs point probe.

The sensitivity of drug concentration to variations in the diffusion coefficient Dis illustrated in Fig 5(a), 5(b). Drug concentrations in both the vitreous humor and the macula exhibit similar trends: increasing D,results in faster early redistribution and more rapid clearance from the (a) vitreous humor and (b) macular region, whereas reducing Dprolongs drug residence time. The predicted half-lives are 0.8, 3.0, and 4.4 days for 10D, 0.1D, and baseline D, respectively, for study 2, following the same overall pattern. These results suggest that diffusion plays the dominant role under the present modeling assumptions. Fig 5(b) further demonstrates close agreement between macular concentrations obtained using point-probe and cylindrical domain extraction, supporting the use of point-probe measurements for the macular concentration results presented throughout this section.

**Fig 6:**
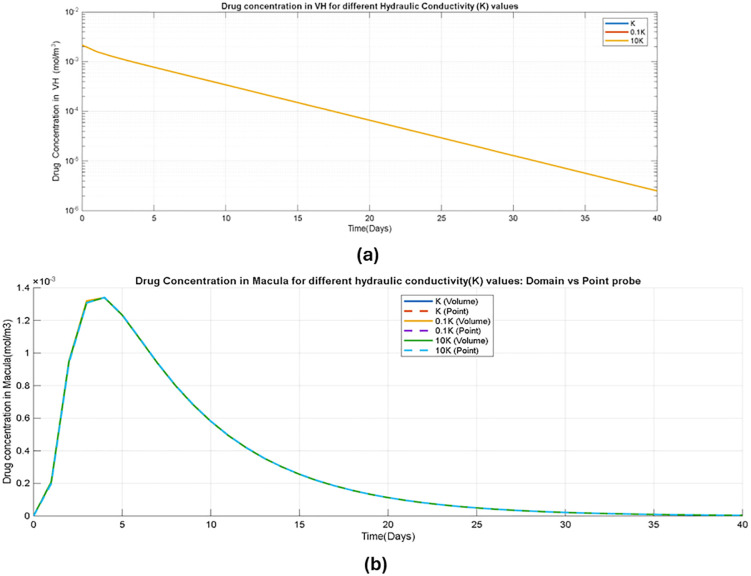
Sensitivity of drug concentration for varying hydraulic conductivity (K) values (a) Drug concentration-time profile in vitreous humor(VH) (b) Drug concentration-time profile in macula using domain vs point probe.

In contrast, varying the hydraulic conductivity Kover the same order-of-magnitude range produces minimal changes in vitreous and macular concentration–time profiles, as shown in Fig 6(a), 6(b). The predicted half-life remains approximately 4 days across all tested Kvalues. This limited sensitivity to hydraulic conductivity suggests that, under physiological conditions, macular drug exposure in the present model is predominantly diffusion-driven rather than convection-dominated. It should be noted, however, that this conclusion is conditioned on the Darcy flow assumptions and boundary conditions adopted here, which inherently produce low convective velocities; models incorporating saccadic eye motion or higher tissue permeability may yield greater convective contributions.

## Discussion

The present study developed a three-dimensional computational framework to investigate the intravitreal pharmacokinetics of ranibizumab and to estimate drug concentration dynamics at the macular region, the primary therapeutic target in neovascular retinal diseases. The model captures key transport mechanisms, including diffusion and convection within the vitreous humor, and evaluates the influence of vitreous structure and elimination pathways.

Due to the limited availability of human *in vivo* concentration data, our present model evaluation was performed by comparing it with published computational studies and reported pharmacokinetic ranges. Therefore, the results should be interpreted as consistent with existing knowledge and physiological plausibility, rather than as definitive empirical validation.

One of the primary challenges in ocular modeling is defining how drugs leave the vitreous chamber. In this study, we utilized two extreme cases: zero-concentration (C = 0) and no-flux to bracket the possible range of ranibizumab clearance. While real ocular tissues possess finite permeability, these scenarios provide essential upper and lower bounds for the drug’s half-life. The predicted half-lives (2.7 ~ 4.4 days) fall within these theoretical limits and match clinical ranges (2.4 ~ 9 days), confirming that the model produces physiologically plausible clearance behavior across both boundary assumptions. As such, the results obtained from these boundary conditions should be interpreted as bounding cases that provide insight into the range of possible pharmacokinetic behaviors.

To estimate the therapeutic duration of ranibizumab, this model utilizes an *in vitro* efficacy threshold of 0.12 µg/mL. While this value represents a clinically significant dose for VEGF neutralization in experimental settings, translating this threshold directly to *in vivo* macular conditions involves inherent uncertainties due to complex physiological factors. Therefore, the estimated duration above this threshold should be interpreted as an approximate indicator of therapeutic potential rather than an exact clinical prediction.

The half-life values reported in this study are derived from volume-averaged vitreous humor concentrations and are primarily used to compare with existing pharmacokinetic studies. In contrast, drug concentration at the macula represents a local measure that directly relates to therapeutic efficacy, particularly for VEGF suppression. These two metrics serve different purposes: while half-life reflects overall drug clearance within the vitreous, macular concentration captures spatially localized drug availability. We acknowledge that while global half-life is the standard reported metric, local decay at the macula is highly sensitive to the initial injection site, as seen in our sensitivity analysis.

Finally, several simplifying assumptions were adopted in this study to enable computational tractability. The vitreous chamber was represented using an idealized spherical geometry, and simulations were conducted under static conditions without incorporating saccadic eye motion. These assumptions may influence drug transport behavior, particularly in partially liquefied vitreous conditions where fluid motion can be more complex. Therefore, the finding that vitreous liquefaction has a limited impact on drug distribution applies specifically to static conditions and may differ under dynamic physiological scenarios.

## Conclusion

By incorporating both diffusion and convection, this study provides a comprehensive analysis of drug ranibizumab kinetics across four physiological scenarios defined by different vitreous states and elimination pathways. Our simulations indicate that partial vitreous liquefaction, by itself, does not markedly alter ranibizumab pharmacokinetics under the static conditions considered in this study. The predicted intravitreal ranibizumab concentration profiles are consistent with previously reported computational and literature-based pharmacokinetic data, supporting the plausibility of the model. The overall drug transport dynamics emerge from a complex interplay of factors (anatomical, physicochemical, and boundary conditions), reflecting the non-linear nature of intraocular pharmacokinetics under the present modeling assumptions. These findings provide a physiologically plausible estimation of intravitreal drug transport behavior; however, they should be interpreted within the limitations of the modeling assumptions, including the use of simplified geometry and the absence of saccadic eye motion.

Accurate knowledge of intraocular drug kinetics is essential for optimizing dosing regimens in neovascular eye diseases. In practice, reported half-lives for intravitreal therapies can vary widely due to differences in experimental methods and patient factors. By using a mathematical modeling approach that can be adapted to different conditions, the framework can be used to explore drug transport behavior and help interpret differences across published studies. Notably, the ranibizumab half-lives predicted by our model (2.7 ~ 4.4 days) fall within the range observed across various clinical and preclinical studies (2.4 ~ 9 days), supporting the physiological relevance of the model. Overall, this computational modeling approach provides useful insights into ranibizumab’s intraocular pharmacokinetics and could aid in optimizing treatment protocols (such as injection intervals) for neovascular retinal diseases. A key limitation of this study is the lack of human *in vivo* concentration data for direct model comparison; future work should aim to incorporate clinical imaging or concentration data to assess further and refine the model. Despite these limitations, our framework can be extended to study other drugs or ocular conditions, underscoring the utility of computational models for understanding and predicting ocular pharmacokinetics.
